# Protocol to quantify and phenotype SARS-CoV-2-specific T cell response using a rapid flow-cytometry-based whole blood assay

**DOI:** 10.1016/j.xpro.2022.101771

**Published:** 2022-09-26

**Authors:** Hygon Mutavhatsindi, Catherine Riou

**Affiliations:** 1 Wellcome Centre for Infectious Disease Research in Africa, Institute of Infectious Disease and Molecular Medicine, University of Cape Town, Observatory 7925, Cape Town, South Africa; 2 Division of Medical Virology, Department of Pathology, University of Cape Town, Observatory 7925, Cape Town, South Africa

**Keywords:** Flow Cytometry/Mass Cytometry, Health Sciences, Immunology, Microbiology

## Abstract

Monitoring antigen-specific T cell frequency, function, and phenotype is essential to assess the host immune response to pathogens or novel vaccines. Here, we describe a rapid and simple *ex vivo* whole blood assay to detect and phenotype the SARS-CoV-2-specific T cell response. We detail steps for whole blood stimulation with SARS-CoV-2 spike peptide and subsequent cell fixation and cryopreservation. We further describe thawing and cell staining steps for flow cytometry analysis. This approach minimizes sample manipulation and has a quick turnaround time.

For complete details on the use and execution of this protocol, please refer to Riou et al. (2021).

## Highlights

• Detection and phenotyping of antigen-specific T cells from whole blood
• Detailed steps for cell fixation, cryopreservation, thawing, and flow cytometry analysis
• Approach requires minimal blood volume and preserves physiological conditions
• Protocol with a quick turnaround time and minimized sample manipulation

## Abstract (editor highlights)

Publisher’s note: Undertaking any experimental protocol requires adherence to local institutional guidelines for laboratory safety and ethics.

## Graphical abstract

**Figure:**
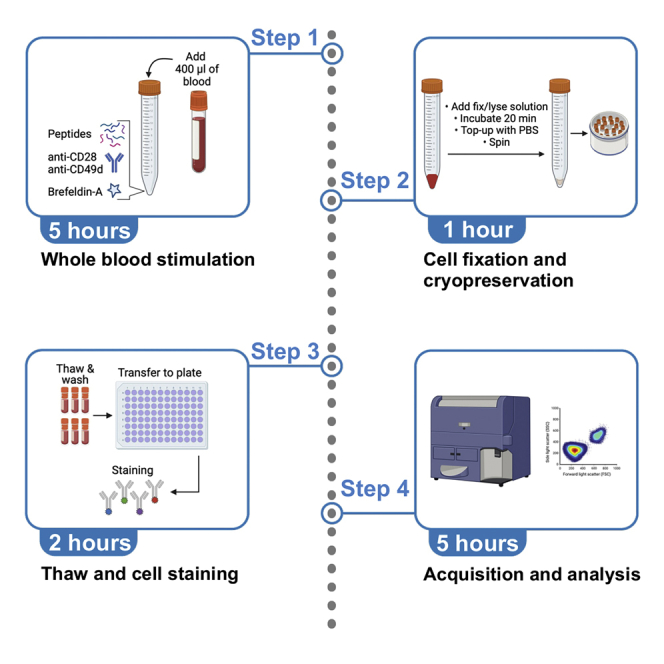

## Before you begin

This protocol provides reagents and specific steps for quantifying and immunophenotyping the SARS-CoV-2 spike-specific T-cell response from whole blood samples. Here, we present an 11-color antibody panel designed to identify IFN-γ, TNF-α, and IL-2 production, memory differentiation markers (CD45RA and CD27), cytotoxic molecules (Granzyme-B and Perforin), and the inhibitory receptor, PD-1. Furthermore, blood stimulation can be performed with peptide pools from other pathogens (examples are given in this protocol using a *Mycobacterium tuberculosis* (Mtb) specific-peptide pool). For the cell staining steps, other antibodies targeting different markers of interest can be included.

Before starting, it is essential to check the configuration of your instrument to ensure compatibility with the reagents used in the proposed panel.

### Institutional permissions (if applicable)

The institution’s Research Ethics Board approval is required when working with human biological material; blood donors provided written informed consent. Additionally, individuals performing this protocol satisfied safety training requirements on the proper handling and disposal of human samples. The whole blood samples used in this study was collected with written informed consent and the approval of the University of Cape Town’s Faculty of Health Sciences Human Research Ethics Committee.

### Reconstitution of PepTivator® SARS-CoV-2 Prot_S complete peptide pool

**Timing: 2 h**

1. Warm-up the vial containing the lyophilized peptide pool (Miltenyi Biotec, cat# 130-127-953) to room temperature.
2. Slowly inject 2 mL of sterile demineralized water with a sterile needle through the center of the rubber plug into the vial containing the lyophilized peptide pool (60 nmol).
3. Vortex the solution to completely dissolve the lyophilized peptide pool (for approximately 20–30 s).
4. Remove the rubber plug and aspirate the stock solution with a pipette.
5. Store in 100 μL aliquots at −80°C.

The concentration of this working stock solution is 50 μg/peptide/mL. Further details can be found in at https://www.miltenyibiotec.com/upload/assets/IM0027281.PDF.

**CRITICAL:** Do not refreeze peptide pool after thawing.

## Key resources table

**Table:** 

REAGENT or RESOURCE	SOURCE	IDENTIFIER
**Antibodies**		

Brilliant Violet 785™ anti-human CD3 antibody (clone: OKT3, dilution:1/83)	BioLegend	Cat# 317330
PE-Cy7™ mouse anti-human CD4 antibody (clone: L200, dilution:1/100)	BD Biosciences	Cat# 560644
Brilliant Violet 510™ anti-human CD8a antibody (clone: RPA-T8, dilution: 1/50)	BioLegend	Cat# 301048
Brilliant Violet 605™ anti-human CD45RA antibody (clone: HI100, dilution: 1/100)	BioLegend	Cat# 304134
CD27 PE-Cy5 anti-human CD27 antibody (clone: 1A4CD27, dilution: 1/50)	Beckman Coulter	Cat# 6607107
PE anti-human CD279 (PD-1) antibody (clone: J105, dilution: 1/100)	eBioscience	Cat# 12-2799-42
BV421™ mouse anti-human Granzyme B antibody (clone: GB11, dilution: 1/167)	BD Biosciences	Cat# 563389
APC anti-human Perforin Antibody (clone: dG9, dilution: 1/72)	BioLegend	Cat# 308112
Alexa Fluor® 700 mouse anti-human IFN-γ antibody (clone: B27, dilution: 1/250)	BD Biosciences	Cat# 557995
FITC anti-human TNF-α antibody (clone: Mab11, dilution: 1/250)	BioLegend	Cat# 502906
PE/Dazzle™ 594 anti-human IL-2 antibody (clone: MQ1-17H12, dilution: 1/72)	BioLegend	Cat# 500344
Purified NA/LE Anti-Human CD28 (clone: CD28.2)	BD Biosciences	Cat# 567116
Purified NA/LE Anti-Human CD49d (clone: 9F10)	BD Biosciences	Cat# 555501

**Biological samples**		

Fresh blood collected in Sodium Heparin (NaHep) tubes	NA	NA

**Chemicals, peptides, and recombinant proteins**		

PepTivator® SARS-CoV-2 Prot_S Complete (GenBank MN908947.3, Protein QHD43416.1)	Miltenyi Biotec	Cat# 130-127-953
Brefeldin A	Sigma-Aldrich	Cat# B7651
1× D-PBS liquid w/o Ca and Mg	Sigma-Aldrich	Cat# D8537
Dimethyl sulfoxide (DMSO)	Sigma-Aldrich	Cat# D5879
Formaldehyde solution (methanol-free, 16%)	Thermo Fisher Scientific	Cat# 28906
HyClone™ Fetal Bovine Serum (FBS)	Merck	Cat# 12107C
RPMI-1640 Medium	Sigma-Aldrich	Cat# D8758

**Critical commercial assays**		

FoxP3/Transcription Factor Staining Buffer Set	EBioscience	Cat# 00-5523-00
Anti-Mouse Ig, κ/ Compensation Particles	BD Biosciences	Cat# 552843
Anti-Rat Ig, κ/ Compensation Particles	BD Biosciences	Cat# 552844
Brilliant Stain Buffer Plus	BD Biosciences	Cat# 566385

**Software and algorithms**		

FlowJo V10.8.1	FlowJo	www.flowjo.com
Graphpad Prism 9.4.0 (453)	GraphPad, LLC	www.graphpad.com

**Other**		

Falcon® 15 mL PP Centrifuge Tube	Corning	Cat# 352096
96-well Clear V-Bottom Microplate	Corning	Cat# 3894
Cryogenic Vials	Corning	Cat# 430661
Falcon® 5 mL Polystyrene Test Tube	Corning	Cat# 352054
CoolCell™ Freezing Container	Corning	Cat# 432006
Polypropylene Microtiter 1.2 mL Tubes	Thermo Fisher Scientific	Cat# 02-681-376

## Materials and equipment

As the quality of flow cytometry data heavily depends on several hardware factors (e.g., laser beam shape and location, choice and quality of optical filters, and sensitivity and resolution of photoelectron detection), careful optimization and calibration of the instrument are necessary. Optimized application settings have been described to obtain maximum resolution of cell populations and consistent results across experiments (Mair and Tyznik, 2019; Perfetto et al., 2012).

All recipes in this section correspond to the volume needed to perform a whole blood assay on 10 donor samples with two conditions (no stimulation and SARS-CoV-2 spike stimulation) using 400 μL of blood per condition (i.e., a total of 20 tubes) and include a 10% overage.

**Table:** Cryopreservation solution

Reagent	Final concentration	Amount
DMSO	10%	2.2 mL
Fetal bovine serum	50%	11 mL
RPMI 1640 Medium	40%	8.8 mL
**Total**	**N/A**	**22 mL**

Prepare fresh for each experiment under sterile condition and keep at 4°C before use.

**Table:** 1× eBioscience Fixation solution

Reagent	Final concentration	Amount
eBioscience Fixation/Permeabilization Concentrate (4×) Cat# 00-5523-00	25%	3.3 mL
eBioscience Fixation/Permeabilization Diluent Cat# 00-5523-00	75%	9.9 mL
**Total**	**N/A**	**13.2 mL**

Prepare fresh for each experiment under sterile condition and keep at room temperature before use.

**Table:** 1× eBioscience Permeabilization solution

Reagent	Final concentration	Amount
eBioscience Permeabilization Buffer (10×) Cat# 00-5523-00	10%	1 mL
Sterile demineralized water	90%	9 mL
**Total**	**N/A**	**10 mL**

Prepare fresh for each experiment under sterile condition and keep at room temperature before use.

**Table:** 1% Formaldehyde solution

Reagent	Final concentration	Amount
Formaldehyde solution (16%)	6.25%	625 μL
PBS	93.75%	9.375 mL
**Total**	**N/A**	**10 mL**

Prepare fresh for each experiment under sterile condition and keep at room temperature in the dark before use.

## Step-by-step method details

### Whole blood stimulation

**Timing: 5 h 30 min (including 5 h incubation time)**

This section describes the steps to stimulate whole blood with SARS-CoV-2 spike peptide.

1. Prepare the stimulation mixes in 1.5 mL Eppendorf tubes labeled appropriately (Negative control and Spike for the unstimulated and SARS-CoV-2 spike condition, respectively) according to Table 1. Start by adding the PBS and then the other components.

**Table 1:** Stimulation mixes

	Stock [ ]	Final [ ]	Vol for 400 μL of blood	Negative control mix (for 10 samples)^a^	Spike mix (for 10 samples)^a^
Anti-CD28	1 mg/mL	1 μg/mL	0.4 μL	4.4 μL	4.4 μL
Anti CD49d	1 mg/mL	1 μg/mL	0.4 μL	4.4 μL	4.4 μL
Brefeldin-A	10 mg/mL	10 μg/mL	0.4 μL	4.4 μL	4.4 μL
Spike peptide pool^b^	0.05 μg/μL	1 μg/mL	8 μL	–	88 μL
PBS (q.s.: 20 μL)	NA	NA	18.8 μL for NS 10.8 μL for Spike	206.8 μL	118.8 μL
Total volume	NA	NA	NA	220 μL	220 μL

a Including a 10% overage.

b The peptide pool used in this protocol does not contain DMSO. If the peptide pool contains DMSO, an equimolar amount of DMSO needs to be added to the negative control mix. q.s.: quantum satis.
2. Add 20 μL of each stimulation mix to the bottom of a 15 mL tube (i.e., 2 tubes per donor, one with the negative control mix and one with the Spike mix).
3. Add 400 μL of whole blood collected in sodium heparin (NaHep) tube to the bottom of each 15 mL tube and mix gently with the pipette to avoid creating bubbles.
4. Incubate at 37°C for 5 h in a benchtop incubator or a water bath.

### Processing of whole blood after stimulation

**Timing: 1 h**

This section describes the steps to simultaneously lyse the red blood cells and fix the leukocytes before cryopreservation.

5. After the 5 h stimulation, vortex gently each tube.
6. Add 1.2 mL (3:1 volume ratio) of 1× eBioscience Fixation solution (see materials and equipment section) to each tube and gently vortex.
7. Incubate for 20 min at room temperature.
8. Add 12 mL of PBS in each tube, close the cap and invert five times.
9. Centrifuge at 400 × *g* for 10 min at room temperature.
10. Discard supernatant and blot the tube edge on absorbent paper to remove excess liquid.
11. Resuspend the pellet by flicking the bottom of the tube manually.
12. Add 1 mL of cryopreserving solution (see materials and equipment section) and gently resuspend the cells.
13. Transfer to appropriately labeled cryovials.
14. Place in a CoolCell™ freezing container and store at −80°C for up to 12 months (see note below).

**Figure 1:**
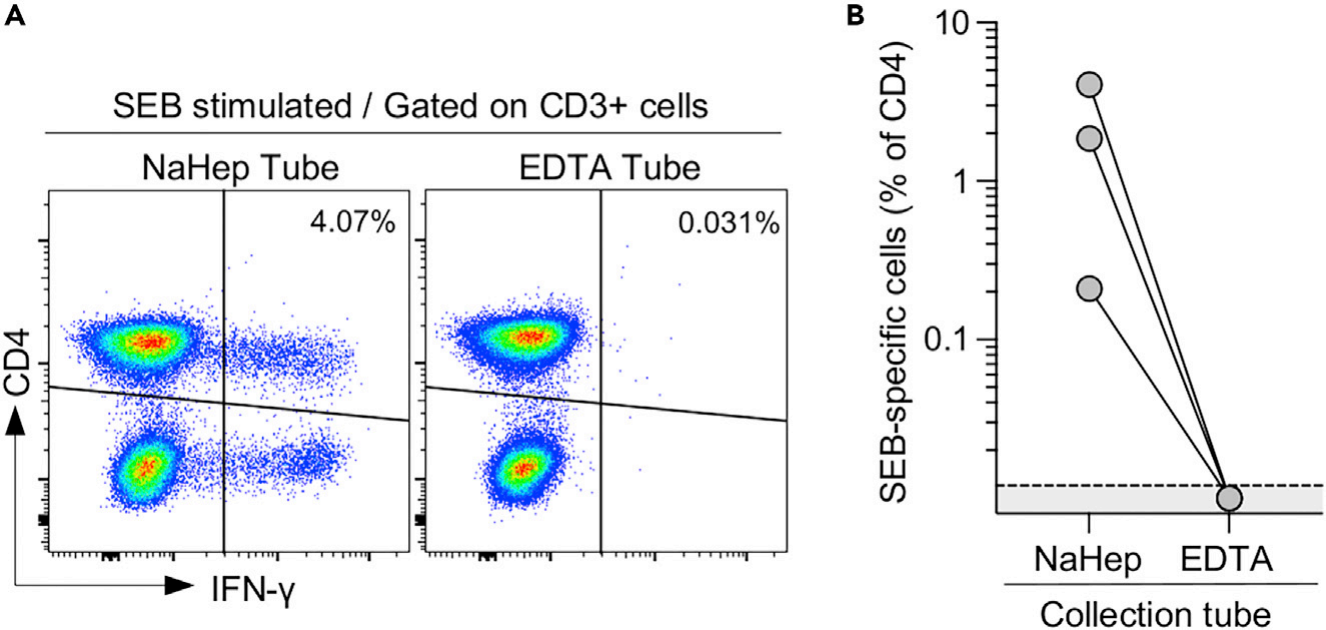
EDTA abrogates IFN-γ production from T cells in response to SEB (A) Comparison of IFN-γ production from CD3+ T cells stimulated for 5 h with SEB (0.5 μg/mL) from NaHep and EDTA blood collection tubes. (B) Frequency of SEB-specific CD4+ T cells from NaHep and EDTA blood collection tubes (n=3). Data are reported after background subtraction (i.e., unstimulated tube).

**Figure 2:**
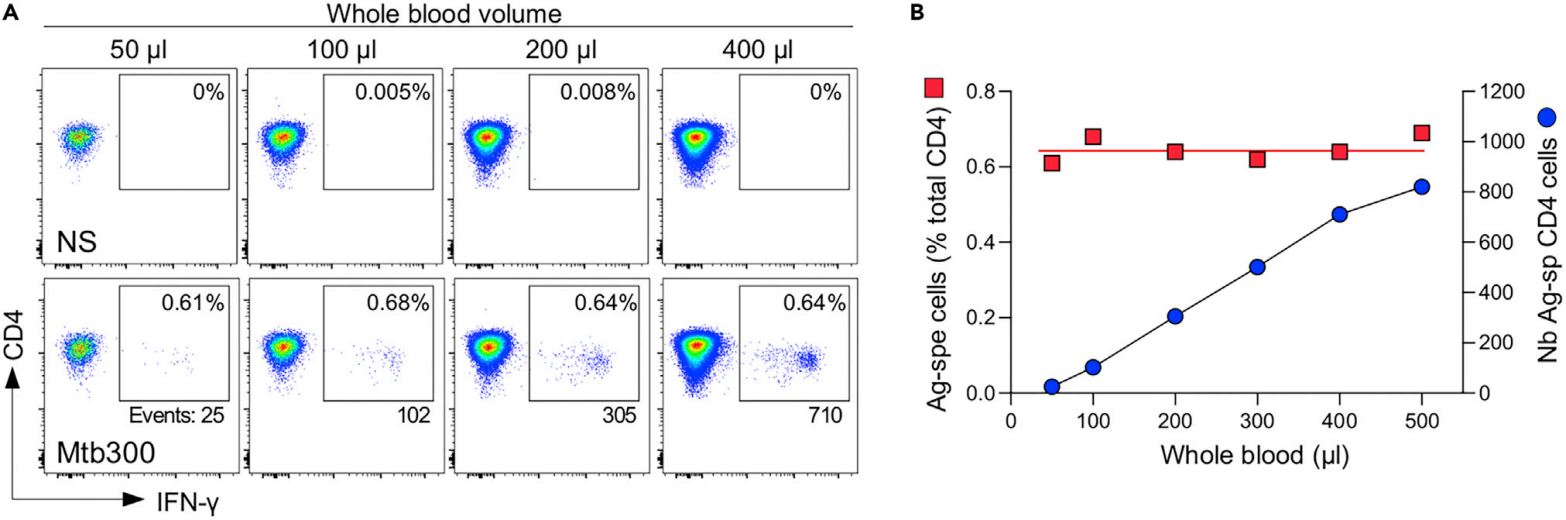
Detection of Mtb-specific IFN-γ+ CD4 T cells in blood volumes ranging from 50 to 500 μL (A) Flow cytometry plots of IFN-γ expression in CD4 T cells from blood stimulated with Mtb300 peptide pool (1 μg/mL) for 5 h. (B) Frequency (red square) and number of events (blue circle) of Mtb-specific IFN-γ + CD4 T cells detected using 50–500 μL of blood (n=1).

**CRITICAL:** Do not use blood collection tubes containing chelating agents such as EDTA. As illustrated in Figure 1, cytokine secretion upon a Staphylococcal enterotoxin B (SEB) stimulation is completely inhibited when the assay is performed using blood collected in EDTA tubes.

***Note:*** The minimal blood volume required for a whole blood assay will depend on the frequency of the cells of interest. Figure 2 shows the assay’s linear range, where blood volume ranging from 50 to 500 μL allows the detection of Mtb-specific CD4+ T cells with a consistent frequency.

***Note:*** It is recommended for blood to be processed within three hours of collection. Delays of more than three hours between blood collection and antigen stimulation have been shown to reduce antigen-stimulated cytokine secretion (Hanekom et al., 2004; Hardy et al., 2021).

***Note:*** If complex antigens such as whole pathogen, pathogen lysate or proteins are used as a stimulus, it is necessary to delay the addition of brefeldin (3–6 h) to allow antigen processing and presentation (Horton et al., 2007).

***Note:*** The cryopreservation step can be skipped, and samples can be stained immediately (see cell staining for flow cytometry section). If samples are cryopreserved, they can be kept at −80°C for up to 12 months without affecting the assay performance (Nemes et al., 2015).

### Thawing of cryopreserved samples

**Timing: 1 h**

This section describes how to thaw the fixed cells in preparation for staining for flow cytometry assessment.

15. Before thawing the samples, prepare 15 mL tubes containing 10 mL of room temperature PBS containing 2% FBS.
16. Thaw cryopreserved samples in a water bath set at 37°C for 1 min 30 s (i.e., when cells are nearly thawed).
17. Transfer cells to 15 mL tube containing 10 mL of PBS 2% FBS.
18. Rinse the vial with 1 mL of PBS containing 2% FBS, and add to the 15 mL tube.
19. Close cap and invert the tube five times.
20. Centrifuge at 400 × *g* for 10 min at room temperature.
21. Discard supernatant and blot the tube edge on absorbent paper to remove excess liquid.
22. Resuspend the pellet by flicking the bottom of the tube manually.
23. Transfer the dead volume (∼70–100 μL) into a 96-V bottom plate.
24. Add 100 μL of 1× eBioscience Permeabilization solution (see materials and equipment section).
25. Incubate for 5 min.
26. Centrifuge at 500 × *g* for 4 min at room temperature.
27. Discard supernatant by flicking the plate (rapidly invert the plate over a waste container and gently tap the plate on a paper towel to remove excess fluid). The cells are now ready for staining.

### Cell staining for flow cytometry

**Timing: 2 h**

This section describes the steps to detect and phenotype SARS-CoV-2 spike-specific T cells. Here, we used an 11-color panel to assess cytokine production (IFN-γ, TNF-α, and IL-2), memory differentiation (CD45RA and CD27), cytotoxic potential (Granzyme-B and Perforin), and the expression of the inhibitory receptor, PD-1.

28. Prepare the fluorophore-conjugated antibody mix in a 1.5 mL Eppendorf tube according to Table 2. Start by adding the 1× eBioscience Permeabilization solution (see materials and equipment section) then the BD Horizon Brilliant Stain Buffer plus and then each antibody.

**Table 2:** Fluorophore-conjugated antibody mix

Target	Fluorophore	Volume per stain	Volume for 20 stains (+10% overage)
IFN-γ	Alexa 700	0.2 μL	4.4 μL
Granzyme-B	BV421	0.3 μL	6.6 μL
TNF-α	FITC	0.4 μL	8.8 μL
PD1	PE	0.5 μL	11.0 μL
CD45RA	BV605	0.5 μL	11.0 μL
CD4	PE-Cy7	0.5 μL	11.0 μL
CD3	BV785	0.6 μL	13.2 μL
IL-2	PE/Dazzle 594	0.7 μL	15.4 μL
Perforin	APC	0.9 μL	19.8 μL
CD27	PE-Cy5	1.0 μL	22.0 μL
CD8	BV510	1.3 μL	28.6 μL
BD Brilliant Stain Buffer Plus		10.0 μL	220 μL
1× eBioscience Permeabilization solution (q.s 50 μL)		33.1 μL	728.2 μL
29. Vortex the antibody mix.
30. Centrifuge the antibody mix at 12,000 rpm (∼8,500 rcf) for 4 min in a mini centrifuge.
31. Store at 4°C in the dark until use.

**CRITICAL:** BD Brilliant Stain Buffer Plus reduces possible compensation artifacts when using multiple Brilliant Violet (BV) reagents in the same flow panel. This buffer is formulated to allow for reduced test volume (10 μL/test), avoiding excessive dilution of the permeabilization buffer during staining.

**CRITICAL:** Keep all antibodies and buffers on ice while preparing the antibody mix.

**CRITICAL:** The centrifugation of the antibody staining mix (step 31) will pellet antibody aggregates that could lead to non-specific staining.

**CRITICAL:** The panel’s performance depends on the sensitivity and setting of the cytometer and reagents. Failing to titrate reagents properly may result in off-scale events or a lack of optimal separation between the negative and positive populations. It is thus critical to properly calibrate the instrument and titrate reagents before the experiment (Maciorowski et al., 2017).

***Note:*** To aid with the discrimination of positive events, one can set up fluorescent minus one (FMO) controls, where the marker of interest is omitted from the staining mix (Roederer, 2002). In this protocol, we strongly recommend using FMO controls for the PD-1 antibody.

***Note:*** Additionally, a “dump” channel can be added (for example CD14 and CD19) to exclude specific cell subsets from the analyses.

32. Add 50 μL of antibody mix to each well.
33. Mix well by pipetting up and down (8–10 times), while avoiding creating excessive bubbles.
34. Incubate for 45 min at room temperature in the dark.
35. Add 170 μL of 1× eBioscience Permeabilization solution.
36. Centrifuge at 500 × *g* for 4 min at room temperature.
37. Discard supernatant by flicking the plate (rapidly invert the plate over a waste container and gently tap the plate on a paper towel to remove excess fluid).
38. Add 200 μL of 1× eBioscience Permeabilization solution and mix well by pipetting up and down (8–10 times).
39. Centrifuge at 500 × *g* for 4 min at room temperature.
40. Discard supernatant by flicking the plate.
41. Add 100 μL of 1% formaldehyde solution and mix well by pipetting up and down (8–10 times).
42. Incubate for 15 min at room temperature in the dark.
43. Add 100 μL of PBS.
44. Centrifuge at 500 × *g* for 4 min at room temperature.
45. Resuspend in 160 μL of PBS and transfer to prelabeled Microtiter tubes and store cells at 4°C protected from light until ready to acquire on the cytometer.

**Figure 3:**
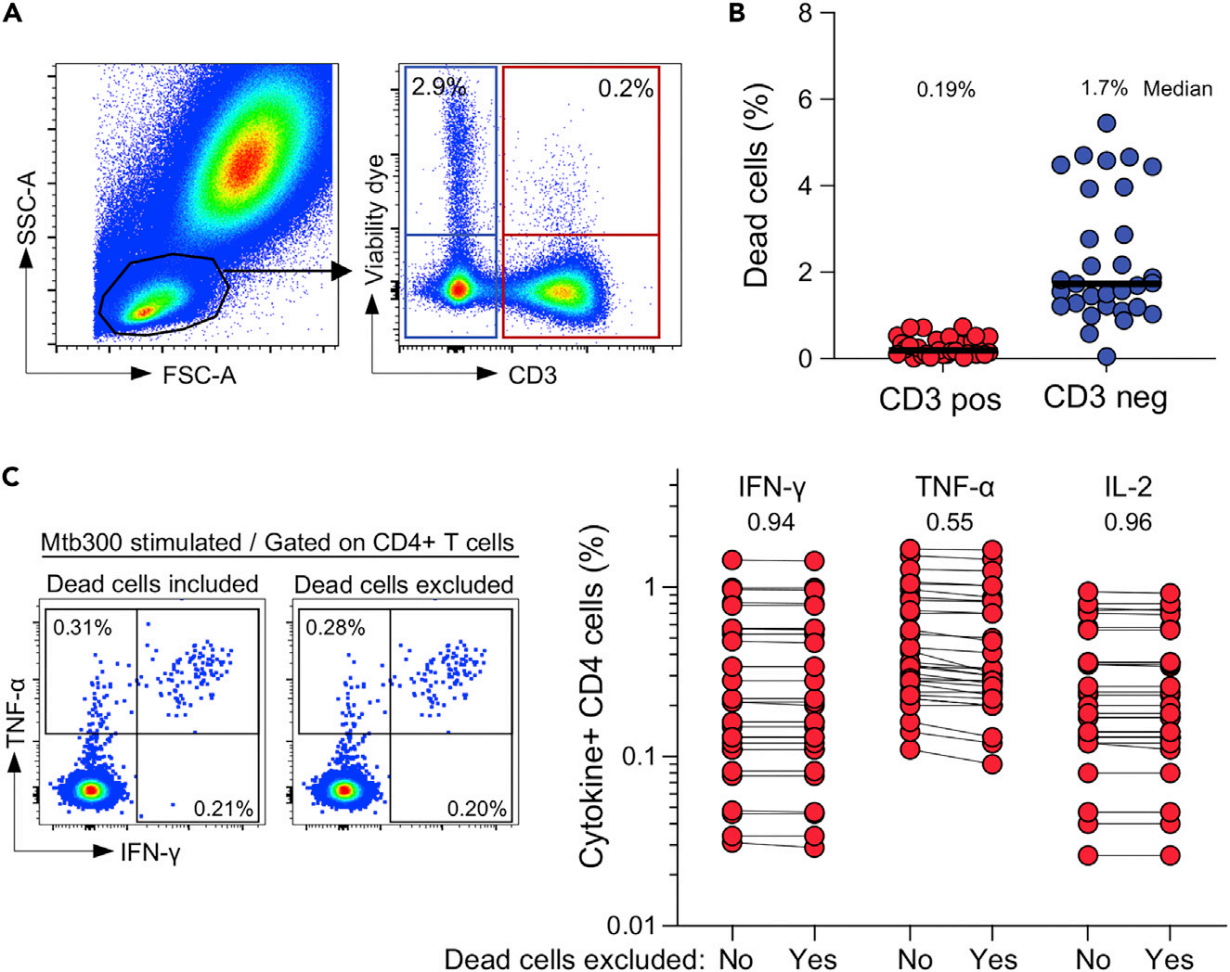
The absence of viability dye in the presented protocol does not significantly affect the assay’s performance (A) Example of viability dye staining of lymphocytes (CD3 positive and CD3 negative cells). (B) Frequencies of dead cells in CD3 positive and CD3 negative lymphocytes (n=30). Bars represent medians. (C) Frequencies of Mtb-specific CD4 T cells producing IFN-γ, TNF-α, or IL-2 with or without exclusion of dead cells (n=30). P values are reported on top of the graph. Satistical comparisons were performed using the Wilcoxon matched-pairs signed rank test.

***Note:*** To ensure the procedure is simple and rapid with minimal perturbation of the samples (i.e., no blood dilution, no erythrocyte lysis prior to stimulation), the usage of a viability dye to differentiate live and dead cells was not included in this protocol. To ensure that this approach does not skew the detection of antigen-specific T-cells, whole blood assays set up to measure Mtb-specific CD4+ T-cell responses were performed in the presence of a viability dye (Riou et al., 2020). Briefly, whole blood was stimulated for 5 h with Mtb peptide, red blood cells were lysed, and live leucocytes were then stained with a viability dye. Cells were then fixed and cryopreserved until batch staining. Data were analyzed to define: 1) the extent of cell death in our assay, and 2) whether the absence of a viability dye affects the quantification of the antigen-specific T-cell response (Figure 3). Our data show that cell death in the T-cell compartment was minimal (median: 0.19%), and the detection of cytokine-producing CD4 T-cells was comparable with or without the exclusion of dead cells. Thus, because blood is processed fresh and the stimulation time before cell fixation is short (5 h), the absence of a viability dye in the staining process does not significantly impact the quality of the results.

### Preparation of flow cytometry compensation controls

**Timing: 30 min**

This section describes how to prepare single stain controls using commercial beads to calculate compensation and correct for spectral overlap between fluorophores.

46. Prepare 14 Polystyrene tubes (5 mL) labeled as describe in Figure 4.

**Figure 4:**
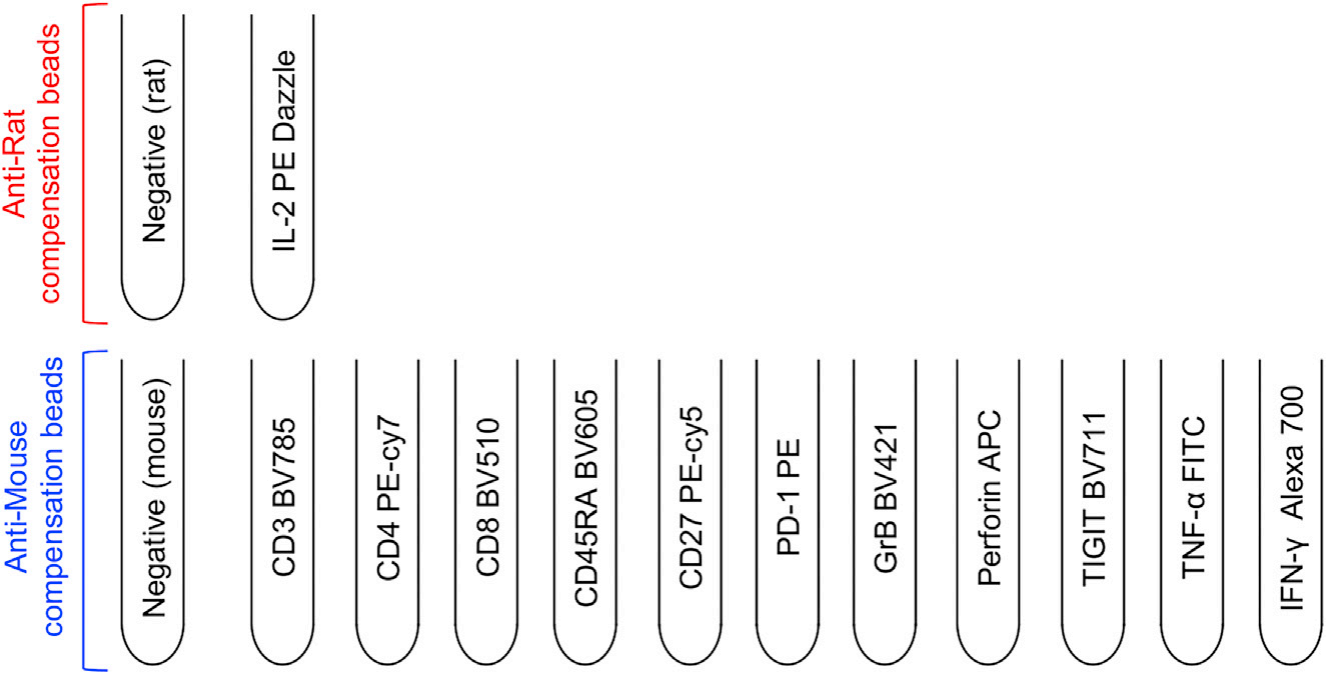
Experimental setup of compensation tubes
47. Add 100 μL of 1× eBioscience Permeabilization solution at the bottom of each compensation tube.
48. Add 15 μL of anti-Rat or anti-Mouse Ig compensation beads at the bottom of the appropriate tubes.
49. Add 1 μL of antibodies to the designated tube.
50. Vortex tubes gently.
51. Incubate for 10 min at room temperature in the dark.
52. Add 2 mL of PBS 2% FBS, vortex the tubes.
53. Centrifuge at 500 g for 10 min at room temperature.
54. Discard supernatant.
55. Resuspend in 200 μL 1% formaldehyde solution.
56. Incubate for 15 min.
57. Add 2 mL of PBS, vortex the tubes.
58. Centrifuge at 500 g for 10 min at room temperature.
59. Discard supernatant.
60. Resuspend in 200 μL of PBS 2% FBS and keep at 4°C protected from light until use.

**CRITICAL:** Since compensation beads precipitate rapidly, vortex the beads thoroughly and regularly before adding them to the 5 mL FACS tubes.

**CRITICAL:** Since compensation control beads are coated with a species-specific antibody, it is important to choose beads that will bind the host species of the fluorochrome-conjugated antibody used. Based on the antibody panel used in this protocol, only one antibody (IL-2 PE dazzle™ 594) is produced in rat, with the remainder being produced in mouse.

**Pause point:** We have not extensively tested saving the samples in the fridge overnight after staining. However, in the case that samples cannot be immediately acquired by the flow cytometer, the cells can be saved for 16–20 h at 4°C, protected from light.

## Expected outcomes

**Figure 5:**
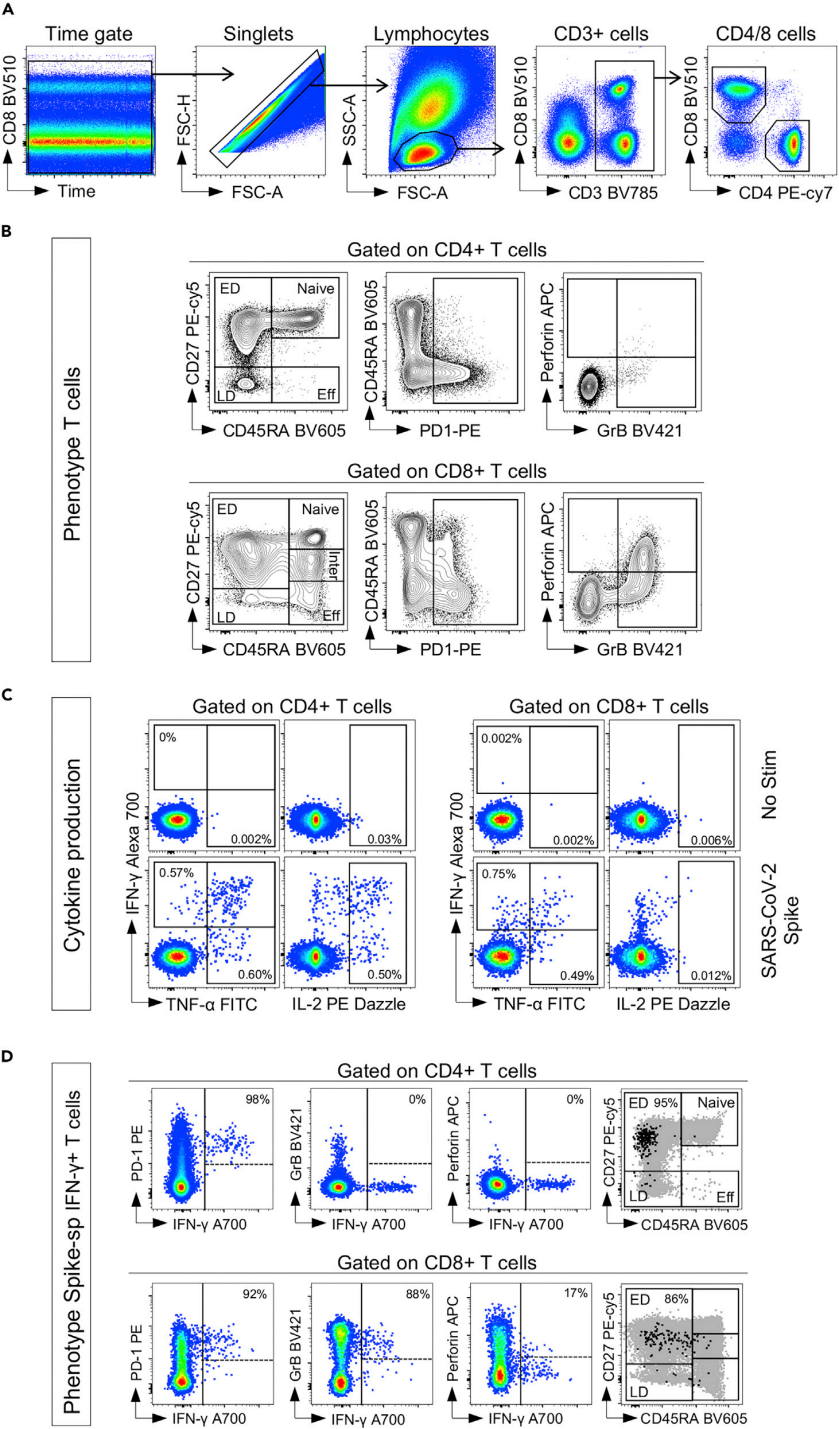
Gating strategy and expected data assessing the frequency and phenotype of SARS-CoV-2 spike-specific T cell response (A) Gating strategy to identify CD4 and CD8 T cells. (B) Phenotype of total CD4 and CD8 T cells. (C) Cytokine expression in CD4 and CD8 T cells in response to SARS-CoV-2 spike peptide pool. (D) Visualization of the phenotype of SARS-CoV-2 spike-specific CD4 and CD8 T cells.

In this section, we show representative results from fully vaccinated healthy donors. The gating strategy, detection of cytokine-producing SARS-CoV-2 spike-specific CD4 and CD8 T cells, and phenotype of Spike-specific IFN-γ+ T cells are presented in Figure 5. The initial gating strategy plots time versus CD8 to inspect the stability of cell acquisition by the cytometer. After gating stable events, single cells are gated by using the FSC-H and FSC-A parameters. Lymphocytes are then identified based on FSC/SSC profile, and CD3+ cells selected. T cells are further divided into CD4+ and CD8+ T cell populations based on the expression of CD4 and CD8, respectively (Figure 5A). Using the expression of CD45RA and CD27, five different memory subsets can be discriminated, namely Naïve (CD45RA+CD27+), early differentiated (ED, CD45RA-CD27+), late differentiated (CD45RA-CD27-), effector (Eff, CD45RA+CD27-) and intermediate memory (Inter: CD45RA-CD27dim, exclusively observed in the CD8 compartment) (Burgers et al., 2009) (Figure 5B). SARS-CoV-2 spike responding T cells were identified based on their ability to produce IFN-γ, TNF-α, or IL-2 (Figure 5C). Finally, the phenotype of cytokine-producing T cells can be visualized by plotting IFN-γ vs. the marker of interest (Figure 5D).

**Figure 6:**
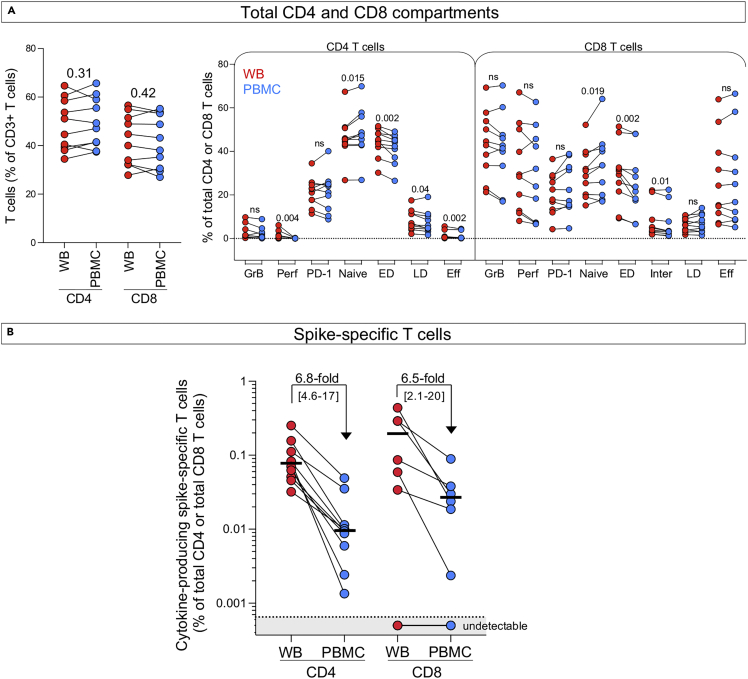
Comparison of the detection of SARS-CoV-2-specific T cells in whole blood and PBMCs PBMC were isolated from the same donors and stimulated using the same conditions (i.e., SARS-CoV-2 Spike peptide pool at 1 μg/mL for 5 h). For PBMC staining, the same antibody panel was used with the addition of a viability marker. PBMC were stained with the following steps: 1) viability marker staining, 2) surface marker staining, 3) fixation, 4) intercellular marker staining and acquired on the same day as the whole blood assay. (A) Comparison of the frequency and phenotype of total CD4 and CD8 T cells between whole blood assay and PBMC. P values are reported on top of the graphs. Satistical comparisons were performed using the Wilcoxon matched-pairs signed rank test. (B) Comparison of the frequency of SARS-CoV-2 spike-specific CD4 and CD8 T-cell responses from paired whole blood (red) and PBMC (blue) (n=10). Median fold change and interquartile ranges are indicated on top. The bar represents the median.

We also compared the performance of the whole blood assay to an intracellular cytokine staining (ICS) assay using paired cryopreserved peripheral blood mononuclear cells (PBMC) and similar experimental conditions (i.e., 5 h stimulation). We used samples obtained from 10 fully vaccinated healthy donors, some of whom experienced a COVID-19 episode. First, we assessed the phenotypic profile of the overall CD4 and CD8 compartments. Figure 6A shows that the frequency of total CD4 and CD8 T cells (expressed as a percentage of total CD3+ cells) is comparable between WB and PBMC. Furthermore, we also compared the frequency of GrB, Perforin, PD-1, and the distribution of memory T cells in the CD4 and CD8 compartments between the whole blood assay and PBMC. Our data show that the overall frequencies of CD4+ and CD8+ T cells and their activation profile were similar between the two assays. the proportion of naïve T cells was slightly lower in the whole blood assay compared to the ICS on PBMC (median: 45% vs 48% for the CD4 compartment and 29.9% vs 33.2% for the CD8 compartment, respectively) as previously reported by Appay et al. (Appay et al., 2006). We then compared the frequency of SARS-CoV-2 spike-specific CD4 T-cell response (producing any measured cytokine: IFN-γ, TNF-α or IL-2) between the whole blood assay and PBMC (Figure 6B). SARS-CoV-2 spike-specific CD4 T-cell response was detectable in all donors with a median frequency of 0.076% (ranging from 0.032% to 0.22%). SARS-CoV-2 spike-specific CD8 responses were detectable in 6 of the 10 donors, with a median frequency of 0.188% (ranging from 0.034% to 0.4%) in responders. The frequency of SARS-CoV-2 spike-specific CD4 and CD8 T cells response were approximately 6-fold higher in the whole blood assay compared to the ICS performed on PBMC, further highlighting the advantage of assessing antigen response using *ex vivo* cell stimulation (Figure 6B).

## Quantification and statistical analysis

**Figure 7:**
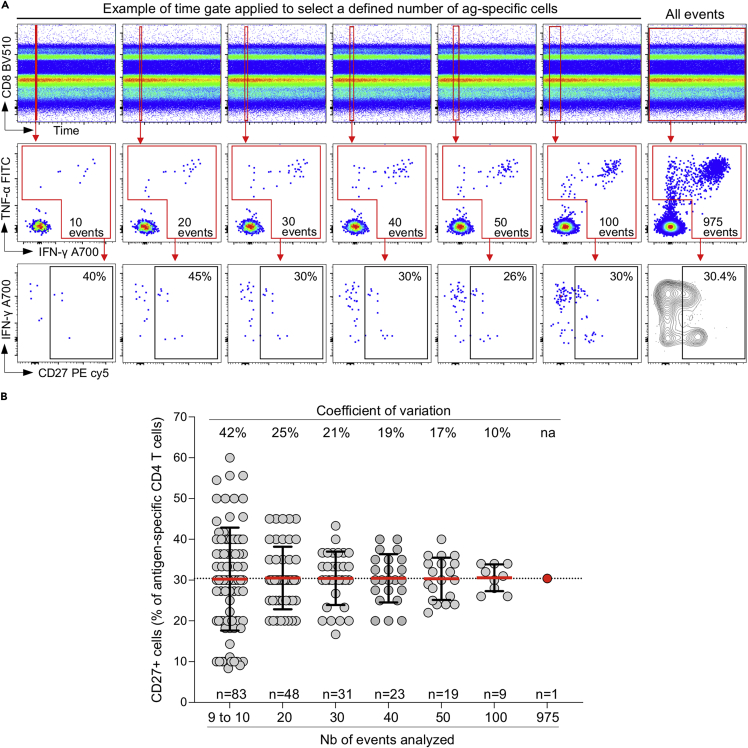
Assessment of the minimal number of cells required to define the phenotype antigen-specific T cells (A) By adjusting the time gate, we performed repeated (non-overlapping) measurements of CD27 expression on antigen-specific T cells (ranging from 10 to 100 cells) to define the variability of the measurement. (B) Summary data showing the expression of CD27 on antigen-specific CD4 T cells from repeated measurements of a defined number of antigen-specific CD4 T cells (10–100). The number of measures done for each sub-group is indicated at the bottom of the graph (n = 83 to n = 9). The coefficients of variation are indicated at the top of the graph. Bars represent the mean with standard deviation.

We used FlowJo v10.7.1 to analyze flow cytometry data. SARS-CoV-2 spike-specific T cells were presented as the percentage of CD8+ or CD4+ T cells that express IFN-γ, TNF-α or IL-2. The gating strategy is shown in Figure 5. A positive response was defined as any cytokine response that was at least twice the background of unstimulated cells and data are reported after background subtraction. To define the phenotype of SARS-CoV-2-specific T cells, a cut-off of 30 events was used to ensure the accuracy of the measurements (Figure 7). Data visualization and statistical analyses were performed using GraphPad Prism v9.1.0.

## Limitations

As cells are fixed immediately after stimulation, some proteins sensitive to fixation (such as chemokine receptors) may not be detectable using this protocol (see troubleshooting, problem 1).

Additionally, this protocol is designed to measure antigen-specific T-cell response using peptides. If complex antigens (such as whole pathogen, pathogen lysate or full-length proteins) are used, further optimizations will be necessary to define the minimal time required for antigen processing and presentation before the addition of the protein trafficking inhibitor (i.e., Brefeldin-A).

Here, SARS-CoV-2-specific T cells were detected based on their ability to secrete three cytokines (IFN-γ, TNF-α, or IL-2). Hence, the panel used is biased towards Th1 CD4 T-cell responses and is likely to underestimate the size of the total spike-specific CD4 T-cell response. This protocol could be adapted to measure agnostic activation markers such as CD137 and CD69 (Altosole et al., 2022). Further optimizations will be necessary to define the appropriate stimulation time to detect such markers.

Lastly, unlike PBMC, the selection of antigen for stimulation needs to be define beforehand.

## Troubleshooting

### Problem 1

Weak or no positive staining of some surface markers.

### Potential solution

Several reasons may result in this problem:

• *Inappropriate antibody titer:* Antibody titration is essential to define the correct concentration of antibody allowing optimal separation between positive and negative populations. Too little antibody will lead to a weak positive signal, and too much antibody can generate an unspecific background signal.
• *Effect of fixation:* Sometimes, even when the material is appropriately fixed, the epitope is not accessible to antibody binding. This issue can be verified by comparing staining between unfixed and fixed cells. Using a different antibody clone may resolve such an issue.

### Problem 2

Weak detection of intracellular markers.

### Potential solution

Saponin-mediated cell permeabilization is a reversible process, it is thus important to dilute the antibody mix in the presence of permeabilization Buffer (1× eBioscience Permeabilization solution in this protocol) (see step 28, Table 2).

### Problem 3

Excessive red blood cells after the simultaneous red blood cell lyse and fixation step (steps 6 and 7).

### Potential solution

A 3:1 ratio of 1× eBioscience fixation solution to blood was sufficient to ensure appropriate lysis of red blood cells. However, this ratio can be increased to 4:1 if necessary.

### Problem 4

Standardization intra- and inter-laboratories.

### Potential solution

As any other flow cytometry-based T cell assays, validation and standardization intra- and inter-laboratories can be a challenge. Standardization of sample processing can be achieved using standard operating procedures and systematic training of operators. It is important to keep the type of anticoagulant in blood collection tubes consistent, ensure that blood in stored and transported at ambient temperature (18°C–22°C). To limit technical variabilities, it is essential that blood samples are processed within 3 h after collection. All commercial reagents used need to be monitored for their expiry date and stored at the appropriate temperature. Keeping record of these essential parameters is thus essential. It is highly recommended to implement log sheets and documents recording the time of blood collection, the time of blood reception, the time when the stimulation was started and stopped, transport temperature, name of the operator and reagent lot number and document any procedure deviations. Such documentation will permit to flag potential samples not meeting experimental requirements. For the flow cytometry staining part of the protocol, it is also recommended to implement positive and negative internal quality controls (QC). The inclusion of unstimulated and stimulated samples generated from a single blood draw in each run can help monitor for batch effects.

## Resource availability

### Lead contact

Further information and requests for resources and reagents should be directed to and will be fulfilled by the lead contact, Catherine Riou (cr.riou@uct.ac.za).

### Materials availability

This study did not generate new unique reagents.

## Data Availability

This study did not generate data set or code.

## References

[bib1] Altosole T., Rotta G., Bornheimer S.J., Fenoglio D. (2022). An optimized flow cytometry protocol for simultaneous detection of T cell activation induced markers and intracellular cytokines: application to SARS-Cov-2 vaccinated individuals. medRxiv.

[bib2] Appay V., Reynard S., Voelter V., Romero P., Speiser D.E., Leyvraz S. (2006). Immuno-monitoring of CD8+ T cells in whole blood versus PBMC samples. J. Immunol. Methods.

[bib3] Burgers W.A., Riou C., Mlotshwa M., Maenetje P., de Assis Rosa D., Brenchley J., Mlisana K., Douek D.C., Koup R., Roederer M. (2009). Association of HIV-specific and total CD8+ T memory phenotypes in subtype C HIV-1 infection with viral set point. J. Immunol..

[bib4] Hanekom W.A., Hughes J., Mavinkurve M., Mendillo M., Watkins M., Gamieldien H., Gelderbloem S.J., Sidibana M., Mansoor N., Davids V. (2004). Novel application of a whole blood intracellular cytokine detection assay to quantitate specific T-cell frequency in field studies. J. Immunol. Methods.

[bib5] Hardy M.Y., Goel G., Russell A.K., Chen Yi Mei S.L.G., Brown G.J.E., Wang S., Szymczak E., Zhang R., Goldstein K.E., Neff K.M. (2021). A sensitive whole blood assay detects antigen-stimulated cytokine release from CD4+ T cells and facilitates immunomonitoring in a phase 2 clinical trial of Nexvax2 in coeliac disease. Front. Immunol..

[bib6] Horton H., Thomas E.P., Stucky J.A., Frank I., Moodie Z., Huang Y., Chiu Y.L., McElrath M.J., De Rosa S.C. (2007). Optimization and validation of an 8-color intracellular cytokine staining (ICS) assay to quantify antigen-specific T cells induced by vaccination. J. Immunol. Methods.

[bib7] Maciorowski Z., Chattopadhyay P.K., Jain P. (2017). Basic multicolor flow cytometry. Curr. Protoc. Immunol..

[bib8] Mair F., Tyznik A.J. (2019). High-dimensional immunophenotyping with fluorescence-based cytometry: a practical guidebook. Methods Mol. Biol..

[bib9] Nemes E., Kagina B.M.N., Smit E., Africa H., Steyn M., Hanekom W.A., Scriba T.J. (2015). Differential leukocyte counting and immunophenotyping in cryopreserved ex vivo whole blood. Cytometry A.

[bib10] Perfetto S.P., Ambrozak D., Nguyen R., Chattopadhyay P.K., Roederer M. (2012). Quality assurance for polychromatic flow cytometry using a suite of calibration beads. Nat. Protoc..

[bib11] Riou C., Du Bruyn E., Ruzive S., Goliath R.T., Lindestam Arlehamn C.S., Sette A., Sher A., Barber D.L., Wilkinson R.J. (2020). Disease extent and anti-tubercular treatment response correlates with Mycobacterium tuberculosis-specific CD4 T-cell phenotype regardless of HIV-1 status. Clin. Transl. Immunol..

[bib12] Riou C., du Bruyn E., Stek C., Daroowala R., Goliath R.T., Abrahams F., Said-Hartley Q., Allwood B.W., Hsiao N.Y., Wilkinson K.A. (2021). Relationship of SARS-CoV-2-specific CD4 response to COVID-19 severity and impact of HIV-1 and tuberculosis coinfection. J. Clin. Invest..

[bib13] Roederer M. (2002). Compensation in flow cytometry. Curr. Protoc. Cytom..

